# The Queen's Hospital, Birmingham

**Published:** 1889-11-09

**Authors:** 


### The Hospital Workshop.

### No. XIV.—THE QUEEN'S HOSPITAL, BIRMINGHAM.

(BY OUR SPECIAL COMMISSIONER).

This hospital is situated in a fine open position in Bath-row, away from the immediate neighbourhood of the factories. It was founded in 1840, incorporated in 1867, and made "free" in 1875; that is to say, subscribers' letters were done away with in that year. A registration fee of one shilling, however, is expected from every patient applying for relief, and at the end of six weeks, should further treatment be needed, another shilling is charged. Nine hundred pounds yearly is added to income from this source, which the opponents of the pay system do not hesitate to say converts the hospital into a huge provident dispensary. That, of course, is an over-statement, but it is not quite clear that the words "free" and "charity" are strictly applicable to an institution that receives money from those who partake of its benefits. If, on the other hand, applicants can afford payment, then an injustice is done to practitioners of the town, who thus lose paying patients.

Of late, at a cost of £26,000, considerable improvements have been made to the hospital, including enlargement and rearrangement of wards, new kitchens, a home for the nurses, servants' dormitories, and other necessary additions. Under the guidance of one of the resident officers your commissioner made a tour of the building.

The out-patient block comprises a large and lofty hall, capable of seating, at a rough guess, about four hundred people. Lying round are the various rooms for surgeons and physicians, the special departments, and the surgeries, where accidents and sudden sickness are treated. The benches in the waiting-hall are arranged on the in-and-out system, with ropes at the sides, so that patients go in at one end and come out at the other, after threading a kind of maze. This plan is common to many hospitals, and has undoubted advantages in preventing confusion, and in keeping strict order of priority, yet the constant movement is apt to be distressing to those who are weak and ill, and presumably most of the applicants are more or less in that condition. Exception is made at the request of any patient, or if he is obviously weak and ill.

A good plan is adopted at many places—namely, that of giving out numbered tickets which settles the turn for consultation beyond the chance of dispute. Work is done here on a large scale; 27,035 out-patients were treated in the year 1888, and of these 14,167 paid the registration fee of one shilling. It appears that 136 applications were rejected on the score of being able to pay for medical advice, and 124 as unfit cases for hospital treatment. The eye department accounted for 2,000 patients, and 641 pairs of spectacles were ordered. In connection with this branch the hospital has six beds divided between the male and female wards. There is a resident obstetric officer, and 364 midwifery cases were attended during the year.

The out-patient block is connected with the main building by a roomy glass corridor, in which male patients are allowed to smoke at certain times of the day.

The chief entrance-hall presents a rather striking appearance. Facing the door is a short flight of steps, which branches off right and left, leading on each side to a well staircase that extends up the whole height of the building. The walls are painted light blue over an olive-green dado, and the whole effect is bright and cheerful. On the ground floor are the board-room and the secretary's offices. On the left opens the glass corridor already mentioned as leading to the out-patient department; a little distance in that direction we reach the accident ward, a homely and comfortable-looking place. On the walls are a few etchings and pictures, while plants and flowers serve to brighten up the tables and windows. The floors are of plain deal, bleached by long scrubbing, the walls are blue, the blinds dark green, and the counterpanes of a Turkey-red blanketty stuff, that adds an artistic touch of warm colouring to the ward. All the bedsteads in this ward have a bent iron rod attached to the frame, and suspending a ring, by which a patient may help to raise himself, and thus relieve the nurses of much of the strain of lifting. Ventilation is effected by means of windows, aided by open fireplaces. Of 413 accidents to men, 183 consisted of broken bones, and twenty-nine of these were fractures of the skull.

Here, in a corner with a screen round the bed, is a young man who three days since fell on his head off the top of a tram. He was brought to the hospital bleeding profusely from the ear, and in an unconscious state. The probability is that, from the spot on the top of the head where he pitched, there was a fracture running down to the base of the skull and passing through the ear. Surgeons now recognise the fact that what makes these injuries fatal is the passage of germs to the interior of the skull, where they set up inflammation of the brain and its membranes. Now, however, the ear is plugged with antiseptic wool, which keeps out germs. In this way many lives are saved that would formerly have been lost; indeed, it was not so very long since that fracture of the base was held to be almost necessarily fatal. A year or two back there was a young man in this ward who fell from a height upon his forehead and outstretched hands, causing fracture of the base and of both arms above the wrist. He subsequently showed the curious symptom of loss of sensation in several of his teeth, but in the end made a perfect recovery.

In one of the male medical wards a boy with phthisis was inhaling antiseptic vapours from an apparatus known familiarly as the "pumpiphone," into which another patient was pumping air. This form of treatment in lung consumption at one time gave great promise of good results, and may yet prove of value in early stages of the disease. There is a large amount of phthisis in Birmingham, but few cases, on account of their protracted nature, can be admitted to the hospitals. The average daily number of occupied beds at Queen's amount to 113, which does not leave much room for the reception of lingering and incurable diseases.

A case in the wards a short time since illustrates forcibly the value of advanced hospital treatment. A man was knocked down by a railway engine, the wheel of which passed over the upper part of his thigh. At the same time he sustained a large scalp wound, but the symptoms did not point clearly to a fracture of the base. His comrades brought him straight to the hospital from the station where the accident happened. Luckily, the surgeon for the day was in the wards at the time, and immediately performed an amputation just below the hip. Delirium set in for a few days, after which he began to mend, and at the end of a week the flaps of the amputation stump were found united. Four weeks later he was ready to leave the hospital. A result of this kind is truly astonishing, and may be attributed to the combined condition of (1) healthy constitution; (2) promptness of treatment; (3) antiseptic surgery; and (4), last but not least, good nursing.

There is no separate ward for children, but eight little cots are placed together at the end of one of the long wards, and shut off by screens. An arrangement of this kind, however, is hardly satisfactory, for the noise of the children cannot fail to disturb the grown-up patients. At the time of our visit a youngster three or four years of age was lying contentedly on his back to let a plaster of Paris splint dry before the fire. His thigh had been broken, but the bone was now firmly knit, and he would be sent home in a day or two.

The hospital stands a little way from the brow of a hill, which shelves off abruptly towards a canal. The grounds at the back are picturesque, and the buildings form a rough quadrangle round what is known as "The Park," in the centre of which stands a single Lombardy poplar. At the far end is the isolation block, formerly a vicarage. Within its ward, the local Kyrle Society has lodged some of its biggest and boldest artistic efforts. Many cases of typhoid fever are treated here, besides a few rare diseases—one of which is called "agarophobia," a nervous affection in which the sufferer is unable to walk along a flat surface, such as a floor.

Skirting the low-lying portion of the grounds next the canal are some official buildings, and among them is a boat-house, where the residents keep a craft that enables them now and then to get clear away into the country—no small boon to men who are tied down to work in a smoky town.

But the newest side of the quadrangle is taken up by the nurses' home. This is a roomy, light, and cheerful place, whither the nurses can come after their work away from the atmosphere of the wards. There are handsome apartments for reading and recreation, and 25 bedrooms arranged to accommodate 37 nurses. This is altogether a move in the right direction, and considering the somewhat straitened condition of finances shows no little courage on the part of the committee.

